# Understanding action concepts from videos and brain activity through subjects’ consensus

**DOI:** 10.1038/s41598-022-23067-2

**Published:** 2022-11-09

**Authors:** Jacopo Cavazza, Waqar Ahmed, Riccardo Volpi, Pietro Morerio, Francesco Bossi, Cesco Willemse, Agnieszka Wykowska, Vittorio Murino

**Affiliations:** 1 Pattern Analysis & Computer Vision (PAVIS), Istituto Italiano di Tecnologia (IIT), Via Enrico Melen 83, 16152 Genova, Italy; 2 IMT School for Advanced Studies Lucca, Piazza San Francesco 19, 55100 Lucca, Italy; 3 Social Cognition in Human-Robot Interaction (S4HRI), Istituto Italiano di Tecnologia (IIT), Via Enrico Melen 83, 16152 Genova, Italy; 4 Department of Computer Science, University of Verona, Strada Le Grazie 15, 37134 Verona, Italy; 5 Naver Labs Europe, 6 Chemin de Maupertuis, Meylan, 38240 Grenoble, France

**Keywords:** Cognitive neuroscience, Computational neuroscience, Computational science, Computer science

## Abstract

In this paper, we investigate brain activity associated with complex visual tasks, showing that electroencephalography (EEG) data can help computer vision in reliably recognizing actions from video footage that is used to stimulate human observers. Notably, we consider not only typical “explicit” video action benchmarks, but also more complex data sequences in which *action concepts* are only referred to, implicitly. To this end, we consider a challenging action recognition benchmark dataset—Moments in Time—whose video sequences do not explicitly visualize actions, but only implicitly refer to them (e.g., fireworks in the sky as an extreme example of “flying”). We employ such videos as stimuli and involve a large sample of subjects to collect a high-definition, multi-modal EEG and video data, designed for understanding action concepts. We discover an agreement among brain activities of different subjects stimulated by the same video footage. We name it as *subjects consensus*, and we design a computational pipeline to transfer knowledge from EEG to video, sharply boosting the recognition performance.

## Introduction

Electroencephalography (EEG) measures the electrical activity patterns induced by the aggregation of excitatory/inhibitory post-synaptic potentials generated in the cerebral cortex. EEG is helpful in registering and monitoring the brain activity, which can then be decoded and used in a variety of scientific, medical and other application domains. For example, from a clinical perspective, there is a well established research direction towards decoding motor imagery (refer to Ref.^1^ for a survey), so that motor neural impulses can be mapped and controlled with the ultimate goal of assisting, augmenting, or repairing human cognitive or sensory-motor functions. Industry is also making efforts in the design of “mind reading” devices in order to let users monitor their well-being^2^, for example, to support meditation or facilitate the execution of daily activities^3–13^.

In the literature, there is a substantial body of works where EEG is utilized in tandem with machine learning and computer vision to recognize useful patterns to face the task of interest. For instance, the recognition of emotions can effectively be addressed through EEG data: stimuli such as natural images or videos can be analyzed in order to figure out the induced emotional state, and measure valence, arousal and dominance factors^14^, while also allowing a finer prediction of happiness, fear or disgust reactions^15^. Furthermore, EEG data are also useful for the general purpose of object categorization, for instance, in the case of the recognition of characters displayed on a screen^16^ or the classification of synthetic/natural images^6,7^.

In this paper, we differ from previous works mainly devoted to read emotions or to decode mental processes related to the classification of what is explicitly visualized in the typical static stimuli, such as characters, digits or objects, and we aim at moving a step forward in the comprehension of the potential associated to EEG data. In particular, we attempt to classify EEG signals related to higher levels of reasoning associated to dynamic stimuli, and specifically devoted to the problem of *understanding the concept behind an action*.

**Figure 1:**
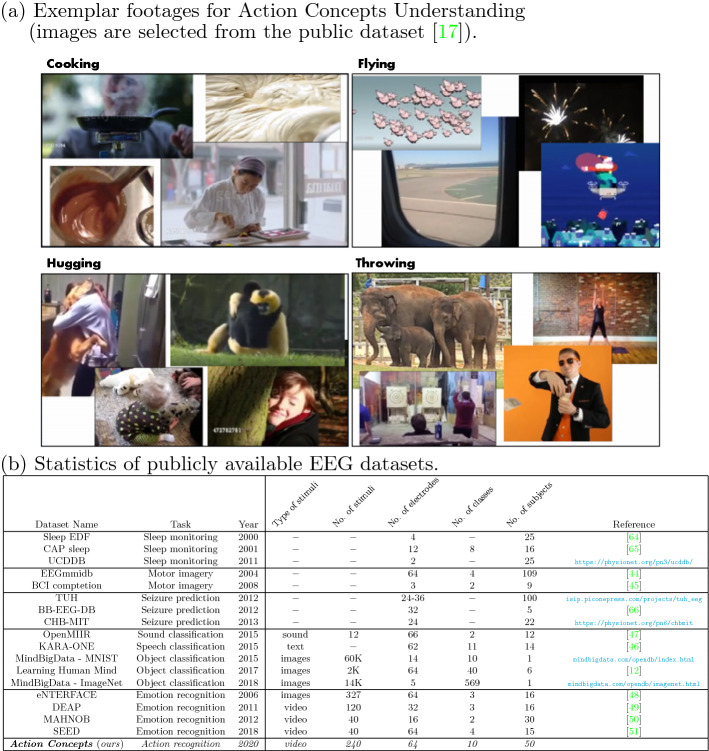
The action concepts dataset. (**a**) Example footages (from the publicly available dataset^17^) related to some of the selected actions for the task of concept understanding, i.e., , recognizing actions that are implied as opposed to be explicitly visualized. The “Cooking” action is represented by the smoke coming out from a pan, the creation of a dough, the mixing of a chocolate cream, or the garnishing of a dessert (*top-left*). The “Flying” action is represented from footages in which pigs fly, fireworks are shot in the sky; alternatively, it is also depicted as a scene in which the panorama is filmed from an airplane window, and as Santa Claus delivering presents (*top-right*). For “Hugging”, unusual cases are considered such as a man hugging his dog, a girl hugging a tree, two gibbons hugging each others, and a baby hugging his toy (*bottom-left*). Similarly, for “Throwing”, elephants can throw sand on their backs, axes can be thrown, a weight can be thrown during a fitness sessions, and eventually money can be thrown in air (*bottom-right*). (**b**) Comparison of existing public benchmarks for EEG data processing, finalized to several applications.

To this end, we built a dataset of EEG recordings acquired from 50 different subjects visually stimulated by videos from the recently designed Moments in Time (MiT) dataset^17^, referring to the following 10 categories of actions: *Cooking*, *Fighting*, *Flying*, *Hugging*, *Kissing*, *Running*, *Shooting*, *Surfing*, *Throwing* and *Walking*. The peculiarity of MiT dataset is the huge intra-class variability, which makes it one of the most challenging datasets that are currently available in computer vision for action recognition^17^. For instance, prototypical videos explicitly visualizing a “flying” action might represent a bird or an airplane in the sky, but we are also considering videos representing such class in an indirect and implicit manner (e.g., the first-person view of the landscape seen from an airplane window), and even extreme and vague cases, such as exploding fireworks (see Fig. 1a). In other words, the problem of understanding action concepts can be defined as classifying an action even when it is not explicitly displayed in a video footage, but, rather, only implied or represented in an abstract sense.

**Figure 2:**
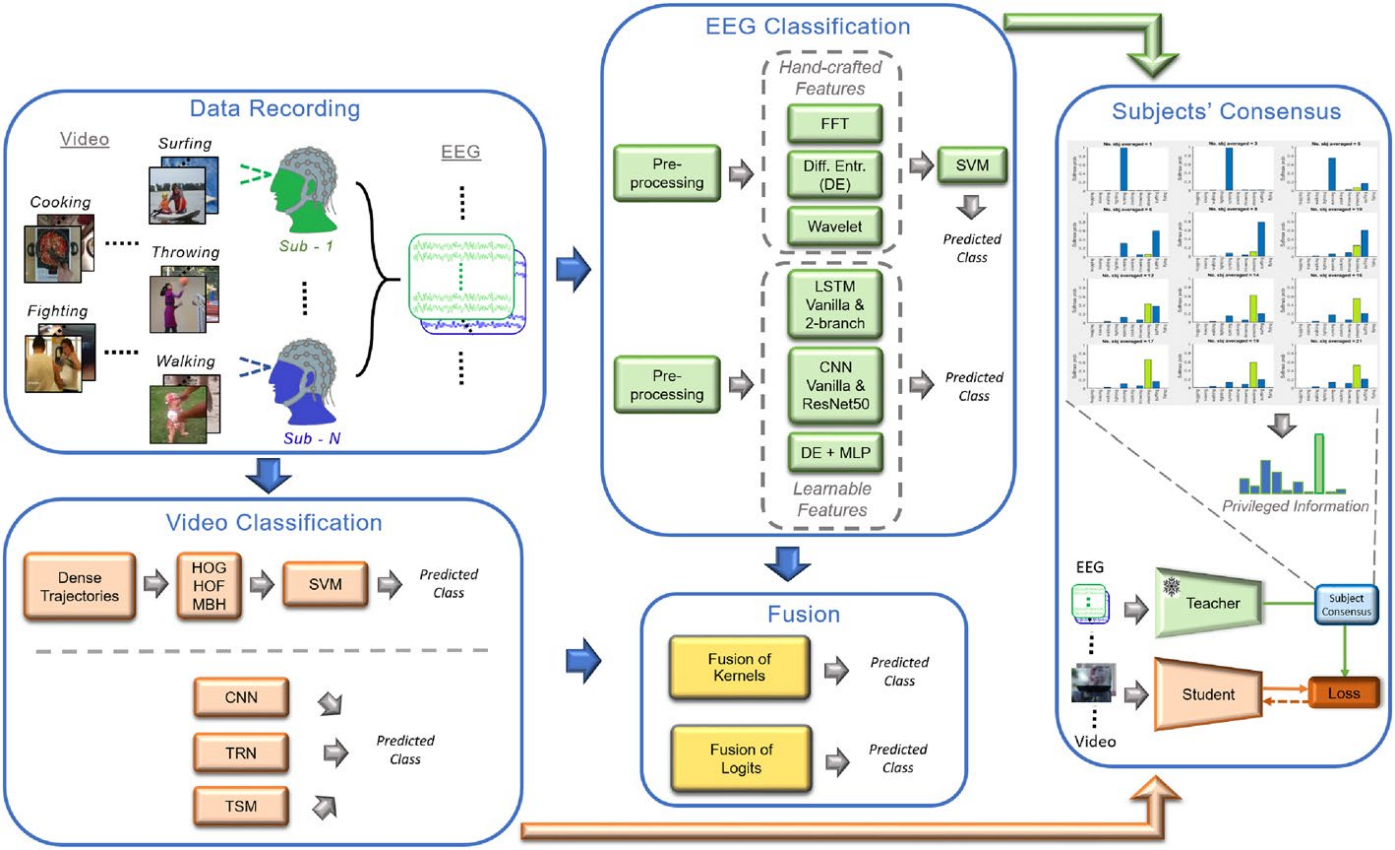
Overview of the several stages of the proposed work. We create the EEG dataset by collecting EEG recordings carrying implicit understanding of each actions’ video footage shown to different subjects (Data Recording). Next, we extract hand-crafted and learnable features to set up various baseline EEG classifiers as *teacher* model(EEG Classification) and video classifiers (Video Classification) as *student* model. Also, we prove through a baseline of fusion methods that the two sources of information are complementary (Fusion). Additionally, we show a strong subject consensus that, when averaging the predictions on models trained on different subjects and tested on the same video footage, the probability of predicting the correct class (green bar) increases. This serves as privileged information used to distill knowledge from EEG to Video model during training (subjects’ consensus).

This paper investigates how brain activity stimulated by video footages can lead to the recognition of action concepts, and formulates the problem as a (multi-modal) classification framework (see Fig. 2). In fact, we posit that EEG is able to capture (some of) the mental processes associated to action recognition and, to the best of our knowledge, our work is the first to demonstrate that understanding action concepts from EEG signals is a solvable and viable problem.

In former computer vision studies in which EEG data is used as an additional modality^6–10,12^, the selected tasks and corresponding benchmarks are relatively easier problems, like character^16^ or object recognition^12^. In principle, both tasks could have been reliably solved without the help of EEG in the sense that, in these works, the performance obtained from EEG data is inferior to that obtained by directly processing the stimuli (e.g., digits or images).

Differently, we claim that EEG is fundamental in our case because visual information is not always reliable for the sake of recognizing action concepts, which can be better understood from brain activity. Through a broad experimental validation considering state-of-the-art methods for video and EEG data processing, we obtained the experimental evidence that the performance achieved by leveraging EEG data is superior to the one of video-only algorithms. Additionally, the two sources of information are complementary, as we proved through a baseline of fusion methods. Therefore, we design a multi-modal computational method to take advantage of EEG data modality in order to boost the performance in classifying action concepts from video data.

Our approach is rooted in the consideration that different people should share some common agreements about which videos are prototypical for which actions, although, we should also account for subjective differences. Therefore, a combination of EEG signals associated with a variety of subjects translates into a peculiar inductive bias having a regularizing effect in filtering out subject-specific nuances. As a consequence, we show that subjects’ consensus boosts the generalization capabilities and favors the development of more accurate video action recognition algorithms, similarly to what happens when adopting an ensemble of models^18–20^).

As a consequence, when averaging together the predictions of subject-specific action classifiers, we register a sharp gain in performance: such a positive effect suggests that there exists an agreement across subjects while predicting actions from EEG data. Our interpretation of such an agreement is that, while the representation associated to a specific action category can be different from subject to subject, low probabilities are in general more likely associated with incorrect classes for a given video. Thus, there is an overall consensus around what classes the video *does not* belong to, and not only a positive consensus on the correct class. The summation of probabilities will eventually result in low probabilities for incorrect classes.

We exploit this finding in a privileged information framework^21^, where the consensus among the subjects yields to a teacher model supported by EEG data. A student model is then distilled by training it with ground-truth action labels as well as soft labels extracted from the teacher. In the end, the consensus that EEG data show when pooling the acquisitions from different subjects is also capable of boosting the performance of two state-of-the-art computer vision models, Temporal Relation Networks^22^ and Temporal Shift Models^23^, conventionally trained with videos only.

### The proposed *action concepts* dataset

We first review publicly available EEG benchmark datasets. Second, we detail the main features of the dataset we acquired, presenting the phase of stimuli selection and subsequent acquisition of EEG recordings from a pool of selected participants.

#### Existing EEG public benchmarks

Electroencephalography (EEG) is a powerful brain activity recording device for a variety of applications. For instance, the problem of recognizing the affective and emotional content of a stimulus was tackled by several works. Event-related potentials can show the connection between a selective processing of emotional stimuli and the activation of motivational systems in the brain^24^. Using gathered data under psychological emotion stimulation experiments, one can successfully train a support vector machine to disambiguate between emotions^25^. In Ref.^26^, EEG data is employed to assess valence and arousal in emotion recall conditions, while comparing different encodings. Facial expressions and EEG are combined for the purpose of affective tags’ generation in a multi-modal approach^27^. EEG was also used to convey patterns related to what makes a movie trailer appealing or not for the audience^28^. Through domain adaptation^29–31^ can cope with the inter-subject differences, and better transfer knowledge across different subjects while recognize emotions evoked by images. The shift from static to dynamical stimulus (videos) in emotion recognition was also investigated in Refs.^32–34^.

EEG capability for creating brain–computer interfaces (BCIs) has also been extensively studied. For instance, in Ref.^3^, a system for rapid image search is devised, whereas EEG and motion capture can be combined in a multi-modal BCI^4^ with the optional usage of deep learning to better fuse the two modalities^5^. Classical computer vision problems, such as object classification in images can be boosted when having access to an ancillary data modality like EEG. For the latter purpose, EEG induced from images shown as stimuli to subjects proved to be effective to boost recognition capabilities, as shown in Refs.^6–10,12,35^. It is notable to mention here that there is an ongoing intense debate about the experimental protocol to be adopted in some of these works^12,35–37^. Since such protocol might resemble the one we adopted, we will discuss these aspects in the “Methods” section. More recently, EEG driven image generation has been tackled in Refs.^35,38^.

However, while EEG was mainly utilized to deal with emotions and BCI applications, it seldom considered dynamic stimuli for the sake of action concept classification. This makes our work pretty unique in the panorama of the multi-modal learning framework.

#### Original characteristics of the dataset

In this work, we exploit EEG to handle dynamical stimuli which consist of 3-s video footages extracted from Moments in Time (MiT) dataset^17^. By design, such dataset was created to guarantee remarkable inter-class and intra-class variations among actions, representing dynamical events at different levels of abstraction (i.e., , “opening” doors, drawers, curtains, presents, eyes, mouths, and even flower petals). While using MiT videos as stimuli for EEG, we attempt to investigate to which extent EEG can convey patterns capable of distinguishing video sequences that, despite their visual diversity, subsume the same category. In fact, very few datasets combine EEG with dynamical stimuli, and fMRI is usually preferred^39–43^).

Specifically, in Fig. 1b, we compare our Action Concepts dataset with those already present in the literature, categorizing the application task (sleep monitoring, motor imagery decoding for BCI, seizure prediction for epilepsy, sound/object classification and emotion recognition). As first peculiar aspect, our dataset ensures high resolution considering a large number of electrodes (64). Second, in terms of key statistical features, existing datasets are extremely unbalanced in terms of number of stimuli vs. number of classes vs. number of subjects considered in the acquisition. In our case, we have (1) a large number of stimuli, (2) a large number of participants (both ensuring high variability within the data), and also (3) more classes, making the classification problem inherently harder. In fact, accounting for many subjects is beneficial when using EEG data since this lends more diversity of the data recorded, and hence more capacity to detect the effect of interest, eventually increasing the reliability of the findings of the study. Overall, unlike the most of existing studies, our proposed dataset is extremely balanced with respect to the above discussed crucial indicators.

Besides, the distinctive feature of our dataset is the targeted application. We do not just recognize what is *explicitly* visualized in the stimuli adopted in the EEG data acquisition, but rather, we attempt to recognize what is *implicitly* visualized in the stimuli themselves. For instance, consider the case of the *cooking* action in Fig. 1a: there are heterogeneous scenarios (involving a smoky pan, a dough, a chocolate cream or even a chef) referring to the same action of interest. Using visual cues to capture the underlying action concept behind “a smoky pan/a dough/chocolate cream/chef” is clearly not enough, and we need to better capitalize from brain decoding mechanisms (captured by EEG activity) that go beyond what is explicitly visualized. To the best of our knowledge, our dataset is the first one designed for recognizing the implicit meaning of visual stimuli.

### Understanding action concepts through subjects’ consensus

When tackling the problem of recognizing activities which are implied from video footages, and not explicitly displayed therein, we must assume that our EEG data could codify a certain degree of subjective interpretation: this is related to the fact that, while processing a given video, each subject will compare it with her/his mental concept for that action. So, it may happen that, for a given subject, her/his mental idea of “flying” will be closer to the one of flying birds, whereas, for another one, it could be that she/he is closer in reasoning towards a centric-view of the panorama visible from an airplane window (see Fig. 1a). This can translate into EEG recordings codifying those subjective traits, ultimately hiding the class-related patterns for that specific action, or making these distinctive patterns very different among subjects.

In other words, each subject is likely to have her/his own biases in understanding activities and, by design, such biases will be captured in EEG signals captured when showing MiT videos to the participants. The fact that, in those videos, the action is not explicitly displayed, but simply implied, will cause EEG signals not just to reflect the perception task of visually parsing the video that has been given as stimulus. Differently, in those EEG signals, we are going to capture the mental processes leading to understand to which extent the video matches the subjects’ personal concept related to a given action. Of course, this depends upon the subject who is watching the video and reasoning on it, but we should also note another important aspect that guided us in the data analysis. Let us make an example: despite a video of fireworks may not be strictly related to “flying” for some individuals, still, the same video will be definitely not categorized as an instance of a “cooking” activity from any subject. In other words, although different subjects may disagree on what is prototypical for a given action (and to what extent), we expect them to agree on *what is not*.

Leveraging this observation, we want to build a computational method that is capable of taking into account several “opinions” about actions’ concepts in order to achieve a more robust action recognition model. To do so, we propose to exploit the *subjects’ consensus*. More specifically, while considering several action classifiers, each trained on a specific subject, we show that, when averaging the predictions of those classifiers, the combined decision score shows a form of consensus in which erroneous predictions are faded out as long as one increases the number of subjects utilized. Remarkably, this happens for testing videos, which were never seen from any of the merged classifiers during training. As a consequence, errors’ reduction translates into sharper predictions for the correct class that, ultimately, yields to better testing classification accuracy.

More specifically, in the subjects’ consensus block of Fig. 2, we consider, as an example, the testing footage of the ground truth class *Surfing* to visualize the predicted probabilities belonging to the 10 classes considered in our study (the probability corresponding to the ground-truth class is highlighted in green). We then ablate on what happens when fixing the testing video and averaging the predictions across a varying number of subjects who watched the video during the experimental acquisition. When considering an increased number of subjects while averaging of predictions, we are taking into account several different opinions. Computationally, this translates into sharper predictions, which are more peaked on the ground-truth class as long as the number of subject increases.

**Figure 3:**
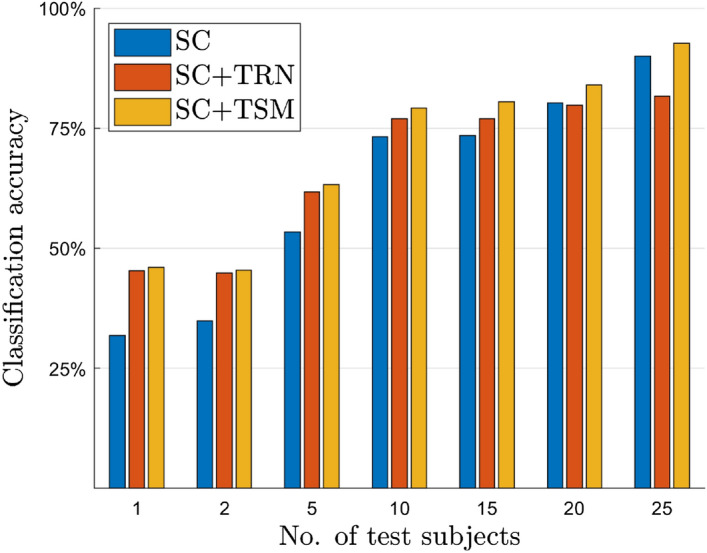
Subjects consensus (SC) as privileged information: EEG boosts video classification. Blue bars correspond to the performance of a DE+MLP (see “Methods” section) model trained on EEG data only: the final classification is done by averaging the softmax prediction over a different number of subjects. Red and yellow bars report the performance of a subject consensus approach on top of the video processing models, in which the averaged prediction over EEG data are furthermore averaged with the prediction of the TRN^22^ and TSM^23^ architectures, respectively, trained on video data. The selected test videos were not used during training, being therefore never seen before from any of the subject-specific predictions that are averaged. Best viewed in colors.

The previous trend is quantitatively confirmed as shown in Fig. 3, in which we evaluate the average video testing accuracy of subjects’ consensus using the predictions of the model that takes advantage of differential entropy (DE) features with a multi-layer perceptron (MLP) classifier (named DE+MLP, technical details available in “Methods” section). Once computed, predictions relative to a single video are averaged across all subjects who watched it. It is interesting to observe that the consensus among subjects is effectively capable of sharply improving the performance, so that the more subjects we consider, the better the performance. Therefore, the effect of the subjects’ consensus keep raising while adding subjects up to 10. Afterwards, we register a sort of plateau in which the performance stabilizes, although, in absolute terms, the best performance is obtained by considering all the subjects. We implement subjects’ consensus on top of the predictions obtained from state-of-the-art computer vision models temporal relation networks (TRN)^22^ and temporal shift models (TSM)^23^—see “Methods” section for the technical descriptions.

In Fig. 3, the effect of such multi-modal fusion is represented by the red and the yellow bars: as one can see, similarly to the case of subjects’ consensus alone (blue bars in the figure), accuracy improves when increasing the number of subjects considered. Also, for a given number of subjects (whose EEG predictions are averaged to get a consensus), adding the video modality to the EEG one is helpful to gain in performance. Interestingly when considering all available subjects that watched a given footage, which is 25 at maximum, the subjects consensus sometimes leads to a superior classification accuracy when using EEG alone if compared to the combination of EEG and videos. We deem this to be a consequence of the difficulty of the video footages which only refer to action implicitly. In this case, by increasing the number of subjects considered, we can better cancel out subjects’ biases. Apparently, the concept related to the ground-truth action to be classified has been unveiled through subjects’ consensus in the EEG data, while it remains rather hidden in the videos.

**Figure 4:**
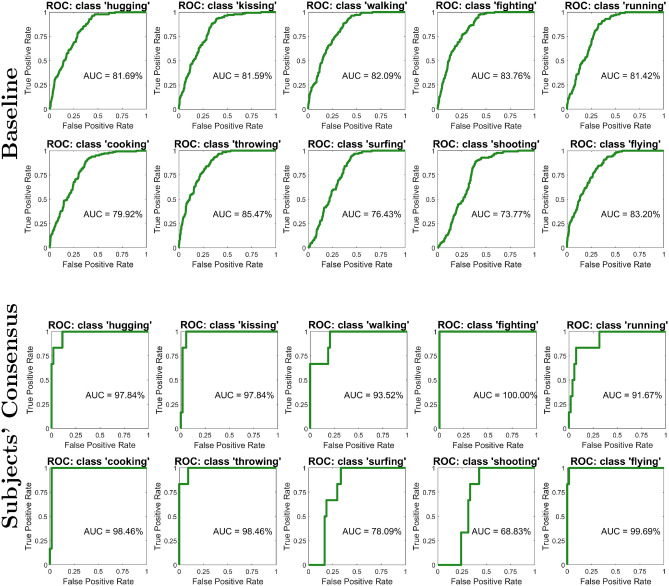
The effect of subject consensus on EEG data. Receiver Operating Characteristic (ROC) curve related to the softmax scores of the multi-layer perceptron (MLP) trained with differential entropy (DE) features. We directly compare the performance, in EEG classification, of the model without model consensus (*top panel*) with the regularizing effect of subjects consensus (*bottom panel*) which improves the action recognition for the video modality.

To better understand the effect of subjects’ consensus, we also provide an evaluation by means of the area under the curve (AUC) for the receiver operating characteristic (ROC) curve. As visible in Fig. 4, with respect to a baseline DE+MLP model trained to perform action recognition from video, the subjects consensus is almost always able to raise the AUC since leveraging the consensus that different subjects seem to exhibit when visually stimulated using the very same video footage. In fact, for the class *Hugging* for example, we get a + 16.15% absolute improvement in the value of the AUC of the ROC and similar improvements were observed for other classes as well, namely: + 16.25% for *Kissing*, + 11.43% for *Walking*, + 16.34% for *Fighting*, + 10.25% for *Running*, + 18.54% for *Cooking*, + 12.99% for *Throwing*, and + 16.49% for *Flying*. In only two cases, we get either a small improvement (+ 1.66% for *Surfing*) or a small drop in performance (− 4.94% for *Shooting*), while overall we got an increment of + 11.51% in average.

Thus, the trend is that the subjects consensus is able to improve the performance over a baseline EEG recognition pipeline without requiring computational changes to the model, but simply aggregating different predictions corresponding to different subjects looking at the same video clip.

In shed of the previous considerations, we are interested in leveraging EEG as a source of privileged information^21^ to boost video classification.

#### Subjects consensus as privileged information

As data modality, EEG needs a specific acquisition setup to be acquired, and, if compared to videos, its portability is clearly inferior. Still, through the potentialities of EEG we are interested in learning an action recognition model (jointly trained on video and EEG data), and then being able to deploy such a model in situations where EEG is not available within input data. In practice, our model is trained on EEG+video, but tested on video data only.

The previous requirements can be framed in the context of learning with privileged information^21^. In order to circumvent the usage of EEG data for inference, we exploit the generalized distillation framework^19–21^ by first training a teacher model to perform action classification on EEG data only. The predictions of this model are computed and smoothed using a softmax temperature^21^. In this manner, we can setup an alternative recognition model (a student) that is trained not only using hard labels, but also considering the teacher’s predictions.

The results of using subjects’ consensus as privileged information are reported in Tables 5 and 6. Therein, we provide the test accuracy averaged over 5 different runs of the same model, also showing the corresponding standard deviation. The results reveal that subject consensus as privileged information offers an improved accuracy of 35.67% as compared to 31.70% accuracy with TRN alone (Table 3). Similarly, the baseline performance of TSM (42.33%) is improved by + 1.33%, reaching 43.66% (Table 3). The overall contribution of subject consensus is summarized in Table 7.

### Conclusions

In this paper, we investigate the problem of understanding action concepts with the support of EEG data for which, with respect to prior work^12,35,44–51^, a higher-level cognitive task is investigated. In particular, we attempted to go beyond the analysis of brain activity which pertains to decoding what individuals merely *watch* in a video stimulus. Rather, we aimed at investigating what the video stimuli actually mean for human subjects considering own interpretation bias for each action concept. We did it empirically by stimulating a pool of 50 subjects through a selection of videos from the Moments in Time dataset^17^ while recording their brain activity by EEG. The peculiarity of the selected videos is that they are designed for action recognition in scenarios where an action is not always explicitly visualized, being only implicit (as illustrated in Fig. 1a as an example). We claim that EEG can go beyond the bare appearance of the stimuli, conveying useful discriminative patterns for the classification of high-level concepts (in this case, related to actions), even if the aforementioned concept is not explicitly visualized, but only (vaguely) implied. Therefore, we have shown to be able to recognize actions’ concepts taking benefit of EEG data captured from visually stimulated subjects. We also performed an extensive experimental analysis of video and EEG data segregately, adopting fusion methods, and exploiting EEG as privileged information for video-based classification models.

To conclude, we speculate that, in these complex scenarios, video classification can be strongly supported by the mental processes that are elicited in a subject who is reasoning about how much the observed footage is prototypical for the ground-truth class. Namely, brain activity captures additional information regarding the subjective interpretation of a specific action and this can also be captured by EEG-based machine learning models which, unlike video-based only methods, can go beyond the mere visual characteristics of the action.

## Methods

### Dataset collection

#### Collecting stimuli: automatic video selection

We exploited an automatic machine learning algorithm to decide which video to include within our analysis. We adopted a ResNet-50 convolutional neural network^52^ pre-trained on ImageNet^52^ to process videos instead of images as done in Ref.^17^. From each video of the Moments in Time training set, 5 random frames are subsampled to fine-tune the ResNet-50’s weights. During inference, a video is classified into one of the 10 classes considering 5 randomly sampled frames followed by a majority voting over the predicted labels. We selected 24 videos per class (240 videos overall) from the MiT validation set by considering only the videos which were correctly classified by the model. Among them, we selected the 12 videos classified with maximal confidence (i.e., , sharpest softmax peak) and the 12 correctly classified with the lowest confidence. In this way, we account for the videos which can be clearly classified by the model as well as the more difficult footages for which the automatic selection stage is less confident (although still managing to achieve a correct inference). In this way, we believe that it is interesting to assess “what is easy/hard to understand” in a comparative scenario between an algorithm (in this case, a neural network) and a pool of human subjects.

#### Selection of the participants

Fifty-three healthy participants (out of which 25 males) were recruited for the experiment (mean age: 23.8 ± 3.71 years). One participant was excluded from the analyses due to technical problems related to data quality. All participants provided written consent before enrolment in this study and were screened for contraindications to EEG. The exclusion criteria included the presence of a history of any neurological or psychiatric disease, use of active drugs, abuse of any drugs (including nicotine and alcohol) as well as any skin condition that could be worsened by the use of the EEG cap. The study was approved by a governative Ethics Committee (Comitato Etico Regione Liguria) and was conducted in accordance with the ethical standards laid out in the 1964 Declaration of Helsinki. All participants had normal or corrected-to-normal vision and were right-handed.

#### EEG acquisition procedure

The participants were asked to sit in a dimly illuminated room, maintaining one meter distance from the screen. There, the EEG cap and EOGs (electrooculography) sensors were put on the head and connected to the EEG amplifier. All the sections of the experiment were run using PsychoPy software^53^. First of all, participants’ resting state activity (with open and closed eyes) was recorded. Subsequently, participants took part in another brief experiment (lasting approximately 15 min), not relevant for this study. Before starting the experiment, participants read the experimental instructions on screen and the experimenter asked for any possible questions or uncertainties. Participants were then presented with a practice part, during which they responded to videos belonging to the category “Eating” (not belonging to the set of actions considered in the actual experiment). After this “practice” phase, participants then moved to the actual experiment, consisting of 5 blocks, one for each category, presented in a random order. Each participant responded to only 5 categories to avoid effects related to the long experiment duration. The categories were counterbalanced across participants, i.e., half of the participants responded to *Cooking*, *Fighting*, *Flying*, *Hugging*, and *Kissing* categories, and the other half responded to *Running*, *Shooting*, *Surfing*, *Throwing*, and *Walking* categories. Each block consisted of the presentation of 24 videos per category in a random order. In the beginning of each block, participants were presented with the action category.

Concerning the “block design” nature of the experiments, a recent debate between Spampinato et al.^12^ and Li et al.^36^ suggest that there is no clear consensus about the best practice on the experimental protocol. While Li et al.^36^ seem to prove that a block design could introduce spurious temporal correlations in EEG acquisition, a further analysis by Spampinato et al.^37^ proved that such correlations are only marginal. Moreover and most important, the best practice regarding the experimental protocol depends on the research question one wants to answer: in our case, we wanted to record the neural activity related to the semantic representation of an action category evoked by looking at an example video of it. To this purpose, the best protocol is the block-based design for the following reasons:

- In order to elicit the semantic representation of a specific action, we had to specify the action category before showing the video. In this way, the participant would develop an a-priori representation of the action and would then compare the video to the semantic representation while watching it^54,55^. After that, the participant could compare each video with her/his representation without having to constantly update the action category that was represented.
- Of course, the action category could be presented before every single video even when visual stimuli were intermixed with no block structure. However, this would imply a continuous update of the action representation and, therefore, a huge cognitive load in carrying out the task. Moreover, if the action category were to switch on a trial-by-trial basis, it would be impossible for the participant to make a direct comparison between examples of the same category.
- Classes and videos are presented in different orders to different subjects, which guarantees there is no temporal correlation between class and tiredness of the subject.
- Last, we intertwine the trials with the oddball orthogonal task^56^, which allows to control for attention to the task and fatigues, further breaking out any possible spurious temporal correlation within the blocks.

#### EEG data recording and pre-processing

EEG data were recorded using 64 Ag-AgCl electrodes of an active electrode system (ActiCap, Brain Products, GmbH, Munich, Germany) referenced to FCz. Horizontal and vertical EOG were recorded from the outer canthi of the eyes and from above and below the observer’s right eye, respectively. The EEG signal was amplified with a BrainAmp amplifier (Brain Products, GmbH), digitised at a 5000 Hz sampling rate for recording. No filters were applied during signal recording. Electrode impedances were kept below 10 k$\Omega$ throughout the experimental procedure. EEG data were analysed using MATLAB™ version R2018a and FieldTrip toolboxes^57^. Data were downsampled to 250 Hz, and a band-pass filter (0.5–100 Hz) and a notch filter (50 Hz) were applied to extract the signal of interest and remove power line noise. Subsequently, data was segmented into epochs (i.e., , trials) from 0 to 5000 ms after the start of each trial. With this segmentation, data from one second before (ISI) and one second after each video were taken into account. Each trial was baseline corrected by removing the values averaged over a period of 1000 ms (from 0 to 1000 ms after the trial started, i.e., , the ISI).

After visual inspection, trials affected by prominent artifacts (such as major muscle movement and electric artifacts) were removed, and bad channels were deleted. However, their values are spherically interpolated using independent component analysis (ICA) so that, effectively, the number of electrodes is always the same across all the different participants. The signal was referenced to the common average of all electrodes^58^, and ICA was applied to remove the remaining artifacts related to eye-blinks, eye movements, and heartbeat. After removing the remaining artifacts, noisy channels were spatially interpolated: at the end of this stage, a total number of 5339 EEG recordings was obtained.

#### Dataset split and train-test protocols

In order to train and validate the computational models described in the next section, the 240 videos were randomly divided in three disjoint splits: Training, Validation and Test, in the proportion 140-40-60, following a consolidated Machine Learning practice. The validation split was employed for hyper-parameter tuning of the models, while results reported in all the tables only refer to the *Test* split. For EEG-only experiments, Training, Validation and Test sets are naturally induced by the Video splits, i.e., EEG stimuli recorded while watching videos in the Video training set constitute the EEG training set and the same holds for Validation and Test.

### Computational methods

#### Methods for EEG classification, hand-crafted features (Table 1, Fig. 2)

Let us define $\mathbf {x}_t$ as a vector concatenating the registered electrical activity of the brain, at a given timestamp *t* in correspondence of all the electrodes ($x^{(n)}$) mounted on the scalp. Over a total of 64 channels, vertical EOG (VEOG) and horizontal EOG (HEOG) are removed as it is usually performed in related studies to get rid of ocular artifacts. Therefore, $\mathbf {x}_t = [x^{(1)}_t, \dots , x^{(n)}_t, \dots x^{(62)}_t] \in \mathbb {R}^{62}$ for each $t = 1, \dots , 750$ since 750 is the number of timestamps (250 timestamps per second are acquired and each MiT video is lasting 3 s). To perform data normalization, each acquired sequence is normalized performing a linear scaling of the range of variability of each channel into the range $[- 1,1]$.

**Table 1:** Performance of hand-crafted features for EEG classification.

	Theta (%)	Alpha (%)	Beta (%)	Gamma (%)
FFT	12.06	13.03	**12.81**	12.66
DE	13.78	13.93	22.62	**28.16**
Wavelet	10.94	11.91	**15.13**	11.16

Bold figures highlight the best frequency range for each method. While FFT and Wavelet show better results in the beta frequency range, DE has a significant boost in the gamma range.

We then compute the *Fast Fourier Transform* (*FFT*) $\{\mathbf {z}_t\}_t$ of the sequence $\{\mathbf {x}_t\}_t$ performing the following computation for each channel:

1 $$\begin{aligned} z^{(n)}_t = \sum _{s = 1}^{750} x^{(n)}_s \exp \left( - s \cdot t \cdot \frac{2 \pi i}{750} \right) , n = 1,\dots , 62. \end{aligned}$$

After computing FFT features, we extract the required frequency windows (theta 5–7 Hz, alpha 8–13 Hz, beta 14–30 Hz, and gamma 31–60 Hz) using the Nyquist’s sampling theorem. We concatenate across different channels and timestamps.

For the *Wavelet transform*, we took advantage of FieldTrip toolbox to compute the mixed and induced power spectrum of Morelet Wavelet function in which we applied an absolute baseline removal strategy in the time window [− 900, − 300] ms before the video starts. To control the number of cycles of the Wavelet function, we perform an adaptive strategy in which we linearly scale the number of cycles (from 3.5 cycles at 2 Hz to 18 cycles at 60 Hz). We downsample the temporal resolution of the input data by a factor of 3 before computing the wavelet function and we perform zero-padding of the input signal by adding 0.2 s before and 0.2 s after it. Again, in correspondence to the selected frequencies of interest, the cut of the computed features is done by using the Nyquist’s sampling frequency, and we concatenate the obtained feature representations into a vectorial embedding representing each instance to be classified.

The entropy of a scalar probability density *f* supported over the Lebesgue measurable space $\mathcal {X}$ is given by

2 $$\begin{aligned} h(f) = - \int _{\mathcal {X}} f(x) \log (f(x)) dx, \end{aligned}$$

and, in the assumption of *f* being distributed as a Gaussian of mean $\mu$ and covariance $\sigma ^2$, it can be easily calculated through the formula^59^:

3 $$\begin{aligned} h(f \sim \mathcal {N}\bigg (\mu ,\sigma ^2)\bigg ) = \frac{1}{2} \log (2 \pi e \sigma ^2). \end{aligned}$$

As shown in Ref.^59^, there is a linkage between the logarithm energy spectrum and the differential entropy of a random variable (being the local error rate *e* constant), which allows us to estimate the *differential entropy* (*DE*) for each of the component of the multivariate time-series $\mathbf {x}_t$ encoding our EEG data. In particular, for each channel—indexed by *n*—we compute the scalar value $h_n$ given by

4 $$\begin{aligned} h_n = \dfrac{1}{2} \log (P_n) + \frac{1}{2} \log \left( \frac{2 \pi e}{750} \right) , \end{aligned}$$

where

5 $$\begin{aligned} P_n = \sum _{t = 1}^{750} \left| \tilde{x}^{(n)}_t \right| ^2, \end{aligned}$$

being $\tilde{x}^{(n)}$ the result of a bandpass filtering of the raw EEG data in correspondence to the frequency window of interest, that is, theta, alpha, beta or gamma.

For either FFT, DE or Wavelet encodings, we apply a zero-centering and a standardization on each feature component. We then train a linear support vector machine (SVM) using the libSVM library and the recommended default parameters choice.

#### Methods for EEG classification, learned features (Table 2, Fig. 2)

We provide a more detailed description on the *neural networks* to learn features from EEG data: the vanilla Convolutional ResNet-based Neural Network (CNN)^52^ , vanilla long-short term memory network (LSTM)^60^ and two-branch LSTM (with attention)^61^. We provide a visualization of their connectivity graph, together with the size of the learnable parameters and other specs: refer to the Supplementary Material, Sect. B and Fig. 1.

The input data is shaped as 62 $\times$ 750 matrix (also denoted as *EEG images*): 62 is the number of channels (once HEOG and VEOG are removed) and 750 is the number of timestamps corresponding to the acquisition time.

**Table 2:** Performance of learnable features for EEG classification.

Recurrent networks		Convolutional networks	
Vanilla LSTM	16.78%	Vanilla CNN	21.80%
Two-branched LSTM	27.04%	EEG images + ResNet-50	33.56%
Two-branched LSTM + attention	28.01%	DE + MLP	39.78%

For the 2D convolutions, we report in brackets a triplet (*h*, *w*, *n*) providing height *h* and weight *w* of the kernels adopted, together with their number *n*. In the case of 1D temporal convolutions, the size of the filters is the very same of the 2D ones, but with the crucial difference that those filters are applied across timestamps (and not slided over a single frame as in 2D convolutions). Dropout layers are paired with the value of the retain probability $p = 0.5$. Rectified Linear Units (ReLU) are occasionally adopted as non-linearity and eventually paired with batch normalization (BN). For the max-pooling operator in the Vanilla CNN, we only perform pooling in time (stride = 50), and we do nothing over the EEG channels. For additional details of the global pooling layer or the (optional) attention module of the two-branch LSTM, refer to the paper^61^.

All three networks have a final softmax classifier, which is responsible for the actual action recognition stage. This is composed of a linear layer, which casts the vectorial embedding produced by the previous layer into a vector *v*, whose size is equal to the number of classes (in our case, 10). The softmax operator eventually produces a probability vector over the classes to be recognized.

In order to create the input *EEG images*, data are pre-processed. At first, FFT is performed on the time series for each trial to estimate the power spectrum of the signal and the three frequency bands of theta (4–7 Hz), alpha (8–13 Hz), and beta (13–30 Hz) are selected. The sum of the squared absolute values within each of the three frequency bands is computed and used as separate measurement for each electrode. The resulting measurements are cast into a 2D image to preserve the spatial structure, while using multiple color channels to represent the spectral dimension. To do so, first, the locations of electrodes are projected from a 3D space onto a 2D sphere using the Polar Projection. Width and height of the image represent the spatial distribution of activities over the cortex and the interpolation is applied to cope with the scattered power measurements over the scalp, and for estimating the values in-between the electrodes over a 32 $\times$ 32 planar square mesh (inducing the pixels). This procedure is repeated for each frequency band of interest, resulting in three topographical activity maps corresponding to each frequency band: red for theta, green for alpha and blue for beta. Then, a number of convolutional neural network architectures have been adopted. The first is a baseline vanilla CNN model. Second, a ResNet-50, pre-trained on ImageNet is directly fed by EEG images. Lastly, an MLP architecture composed by a hidden layer of size 248 with rectifield linear units as non-linearities and dropout with rate 0.5, is fed with DE features (named DE+MLP).

As a general consideration (holding for both EEG and video classification methods), it is important to note that, when dealing with a deep machine learning model, it is always the case that the model learns correlations or generic associations between data-learnt features and labels. However, these associations are not necessarily interpretable since features are the abstract numerical components of the tensors automatically learned by deep layers of the neural networks, for which a human interpretation is hardly possible. Further details are available in the Supplementary Material, Sect. C.

**Table 3:** Performance for video classification only.

Dense trajectories: HOG	18.64%	ResNet-50	29.33%
Dense trajectories: HOF	16.95%	Temporal residual networks ($N=4$)	28.30%
Dense trajectories: MBHx	23.33%	Temporal residual networks ($N=8$)	31.70%
Dense trajectories: MBHy	26.67%	Temporal shift models	42.33%

#### Methods for video classification (Table 3, Fig. 2)

We tested three methods for Video Action classification:

1. We take advantage of a spatio-temporal interest point detector capable to retrieve “corners” in space+time, which represent voxels characterized by a major dynamical variation, i.e. *dense trajectories* (*DT*)^62^. The spatio-temporal interest points are found from optical flow and they are tracked for a number *L* of consecutive frames (we use the default parameter $L = 15$). In correspondence of each of the previous trajectories, a warped volume $\mathcal {V}$ is defined so that each trajectory is always at the center of a vertical slice of $\mathcal {V}$ itself (we exploit the default parameter to define the dimension of the slice). From each volume $\mathcal {V}$, several histogram features are computed: histograms of oriented gradients (HOG), histograms of oriented optical flow (HOF) and motion boundary histograms (MBH), which are particularly useful to handle cases in which the camera moves with respect to the scene captured in the video (MBHx and MBHy for either horizontal or vertical displacement). For each of these classes of histogram descriptors, many of them are extracted from a single video footage, and the aggregation process into a fixed vectorial embedding with which the video can be represented is done by means of bag-of-words pooling (using a dictionary of 1000 codewords extracted by means of *K*-means clustering, $K = 1000$). At the end of this process, a $\chi ^2$ kernelized SVM is trained and responsible for the final video classification. To do so, we took advantage of libSVM library, using default parameters.
2. We exploited *Temporal Relation Network* (*TRN*)^22^, which is an action recognition method devised to simultaneously model several short and long range temporal relations between sparsely sampled frames. Given a video *V*, composed of *n* selected ordered frames $f_1,f_2,...,f_n$, 2-frame temporal relations $T_2(V)$ are defined as
  $$\begin{aligned} T_2(V) = h^{(2)}_\phi \bigg (\sum _{i<j}^{}g^{(2)}_\theta (f_i,f_j)\bigg ), \end{aligned}$$
  and 3-frame temporal relations as
  $$\begin{aligned} T_3(V) = h^{(2)}_\phi \bigg (\sum _{i<j<3}^{}g^{(3)}_\theta (f_i,f_j,f_k)\bigg ). \end{aligned}$$
  Analogous definition can be expressed for longer-term temporal relationships $T_4(V)$, ..., $T_N(V)$. In the previous formulas, $f_i$ represents the extracted features of *i* *th* frame, and $h_\phi ^{(d)}$ and $g_\theta ^{(d)}$ are single-hidden layer neural networks, capturing different timescales (i.e.,  different number of frames) $d=1,\dots ,N$. The overall optimization objective $\mathcal {L}$ for the video *V* is $\mathcal {L}(V) = T_2(V) + T_3(V)... + T_N(V)$ which is optimized via gradient descent with respect to the parameters of the networks $h_\phi ^{(d)}$ and $g_\theta ^{(d)}$. In our experiments, using the TRN architecture, we adopted the BN-Inception model^63^ pre-trained on ImageNet to extract frame-level feature $f_i$. Also, the hyper-parameter *N* in the equation for $\mathcal {L}$ above is selected using prescribed values in Ref.^22^, alternately fixing $N=4$ and $N=8$ to capture medium and long term dependencies in time. Default training strategies of batch normalization and dropout after global pooling are also used.
3. In *temporal shift models* (*TSM*), standard convolutional neural network baseline architectures (here, we used ResNet-50 as in Ref.^23^) are extended to handle temporal data by introducing, in addition to frame-wise 2D convolutions, 1D temporal convolution among temporal shifted version of the input video across time frames. For instance, given an input video of $I_t$ frames indexed over a timestamps *t*, in addition to 2D convolutions acting on $I_t$ for each *t* in parallel, the temporal shift model also computes a 1D temporal shifted convolutions according to the formula $w_1 I_{t-1} + w_2 I_t + w_3 I_{t+1}$, in the case of a temporal kernel of length 3. Note that the weights of the temporal kernel for shifted convolutions are shared across different shifted version of the input video.

#### Fusion methods for joint EEG and video classification (Table 4, Fig. 2)

We adopted two types of fusion approaches. In the *kernel fusion* method that we consider in the paper, we took advantage of multiple Gram matrices, each of them computed from a single descriptor out of the many we considered: MBHx and MBHy features (encoded with Bag of Features) extracted with dense trajectories, the hidden representation of the MLP fed with DE features, and the feature vector produced by the last average pooling layer of ResNet-50 fed with EEG images. For each feature, we computed a linear kernel and the resulting Gram matrices are averaged and fed to a Support Vector Machine for classification. To train this kernelized SVM machine, we took advantage of libSVM library using default parameters.

**Table 4:** Performance of the fusion methods.

Kernel fusion	46.14%
Fusion of logits	45.47%

We have also explored the so-called “late” *fusion of logits* approach. Specifically, we selected the best video model (TSM) and the best model for EEG (MLP+DE, i.e., MLP fed with DE features). In each model, the input vector to a softmax operator is extracted, averaged together and the final classification performance is computed by arg-maxing over it.

#### Subject consensus: technical details (Fig. 2)

We implemented subjects’ consensus by considering the baseline EEG model consisting in DE features fed into a multi-layer perceptron MLP. In particular, we took advantage of the logits of that model (that is, the vectorial representation which is normalized into a probability density by applying a softmax operator). When a specific video footage needs to be classified, we considered all the subjects to which that footage was presented as stimulus during the database acquisition. In order to classify such footage, we compute the logits of the DE+MLP that processed all the available EEG recordings (belonging to different subjects) corresponding to that footage. Afterwards, we average the logits and apply softmax for visualization purpose. In fact, such operation produces a probability density, indexed over the selected 10 classes, showing the most likely prediction according to the model.

The previous requirements can be framed in the context of learning with privileged information^21^. Specifically, within the task of predicting $y_i$ given $x_i$, $i=1,\dots ,n$, privileged information leverages additional information $x_i^\prime$ about the example $(x_i,y_i)$. In our case, $x_i$ will correspond to a video footage, while $x'_i$ will represent an EEG recording of a given subjects watching the same footage as stimulus. In order to circumvent the usage of EEG data for inference, we exploit the generalized distillation framework^19–21^ by first training a teacher model $f_t$ to perform action classification on EEG data $x_i'$ only. Second, we compute the predictions $s_i = \sigma (f_t(x_i')/T)$ using a temperature parameter *T* in order to have a smoothing effect and enhance commonalities and differences between classes to be discriminated, which is the actual potential of using soft labels^21^. Then, we train a student model $f_s$ *using video data only*, by minimizing the following loss:

6 $$\begin{aligned} \frac{1}{n} \sum _{i=1}^{n}\bigg [(1-\lambda )l(y_i,\sigma (f(x_i))) + \lambda l(s_i,\sigma (f(x_i))\bigg ], \end{aligned}$$

where the imitation factor $\lambda \in [0,1]$ controls the balance between predicted soft labels $s_i$ and ground-truth hard annotations $y_i$. Tables 5, 6, 7 summarize the results of using subject consensus as privileged information.

**Table 5:** Temporal relation networks (TRN)^22^ with subjects consensus.

	$\lambda = 0.25$		$\lambda = 0.50$		$\lambda = 0.75$		$\lambda = 1.00$	
*T*	Acc (%)	Std (%)	Acc (%)	Std (%)	Acc (%)	Std (%)	Acc (%)	Std (%)
1	30.00	2.35	32.67	2.53	32.34	1.49	34.33	3.84
2	31.67	2.04	33.33	1.18	33.33	2.04	31.67	4.72
5	33.33	2.64	34.33	1.49	34.67	2.17	35.33	2.47
10	33.00	2.17	35.67	1.49	34.67	2.74	31.67	3.12
20	33.33	2.36	31.67	1.18	32.67	0.91	22.00	1.82
50	33.33	2.64	32.33	0.91	34.33	0.91	10.33	0.75

**Table 6:** Temporal shift models (TSM)^23^ with subjects consensus.

	$\lambda = 0.25$		$\lambda = 0.50$		$\lambda = 0.75$		$\lambda = 1.00$	
*T*	Acc (%)	Std (%)	Acc (%)	Std (%)	Acc (%)	Std (%)	Acc (%)	Std (%)
1	43.33	1.66	43.00	1.39	42.33	1.90	42.66	0.91
2	43.66	1.39	43.00	1.39	40.66	2.23	42.99	0.74
5	40.66	1.90	36.33	1.39	33.00	1.39	23.66	6.49
10	38.99	0.91	32.33	0.91	30.33	1.39	22.66	3.83
20	38.00	1.39	31.33	1.39	27.00	0.74	19.66	1.82
50	37.66	0.91	31.00	0.91	27.66	0.91	15.66	0.91

**Table 7:** Highlights of the best achieved performance across all data modalities.

EEG only	Video only	Subjects’ consensus	Fusion
39.78%	TRN: 31.70%	31.70% $\rightarrow$ 35.67%	46.14%
	TSM: 42.33%	42.33% $\rightarrow$ 43.66%	

Subjects’ consensus refers to VIDEO only performance, where models are trained with the additional supervision provided by the consensus predictions inferred from the EEG of the multiple subjects who watched a specific video. Fusion refers to explicit EEG+VIDEO fusion and is thus an upper bound for model performance.

## Supplementary Information

Supplementary Information.

## Data Availability

The dataset collected analysed during the current study is available from the corresponding author on reasonable request and will be made public upon paper acceptance.

## References

[1] Tiwari, N., Edla, D. R., Dodia, S. & Bablani, A. A comprehensive survey. In *Brain Computer Interface: Biologically Inspired Cognitive Architectures* (2018).

[2] Hou, X., Liu, Y., Sourina, O., Tan, Y. R. E., Wang, L. & Mueller-Wittig, W. Eeg based stress monitoring. In *2015 IEEE International Conference on Systems, Man, and Cybernetics* (2015).

[3] Gerson AD, Parra LC, Sajda P (2006). Cortically coupled computer vision for rapid image search. IEEE Trans. Neural Syst. Rehabil. Eng..

[4] Jungnickel E, Gramann K (2016). Mobile brain/body imaging (mobi) of physical interaction with dynamically moving objects. Front. Hum. Neurosci..

[5] Pérez-Benítez, J. L., Pérez-Benítez, J. A. & Espina-Hernández, J. H. Development of a brain computer interface using multi-frequency visual stimulation and deep neural networks. In *International Conference on Electronics, Communications and Computers (CONIELECOMP)*, 18–24 (2018).

[6] Kapoor, A., Tan, D., Shenoy, P. & Horvitz, E. Complementary computing for visual tasks: Meshing computer vision with human visual processing. In *2008 8th IEEE International Conference on Automatic Face & Gesture Recognition*, 1–7 (2008).

[7] Kapoor, A., Shenoy, P. & Tan, D. Combining brain computer interfaces with vision for object categorization. In *2008 IEEE Conference on Computer Vision and Pattern Recognition*, 1–8 (2008).

[8] Omedes, J., Iturrate, I., Montesano, L. & Minguez, J. Using frequency-domain features for the generalization of eeg error-related potentials among different tasks. In *2013 35th Annual International Conference of the IEEE Engineering in Medicine and Biology Society (EMBC)*, 5263–5266 (IEEE, 2013). 10.1109/EMBC.2013.6610736 24110923

[9] Bashivan, P., Rish, I., Yeasin, M. & Codella, N. Learning representations from eeg with deep recurrent-convolutional neural networks. *International Conference on Learning Representations (ICLR)* (2016).

[10] Fatima, S. & Kamboh, A. M. Decoding brain cognitive activity across subjects using multimodal m/eeg neuroimaging. In *2017 39th Annual International Conference of the IEEE Engineering in Medicine and Biology Society (EMBC)*, 3224–3227 (IEEE, 2017). 10.1109/EMBC.2017.8037543 29060584

[11] Tseng Y-H, Tamura K, Okamoto T (2021). Neurofeedback training improves episodic and semantic long-term memory performance. Sci. Rep..

[12] Spampinato, C. *et al*. Deep learning human mind for automated visual classification. In *2017 IEEE Conference on Computer Vision and Pattern Recognition (CVPR)* (2017).

[13] Di Liberto GM (2021). Robust anticipation of continuous steering actions from electroencephalographic data during simulated driving. Sci. Rep..

[14] Abadi MK, Subramanian R, Kia SM, Avesani P, Patras I, Sebe N (2015). Decaf: Meg-based multimodal database for decoding affective physiological responses. IEEE Trans. Affect. Comput..

[15] Westner BU, Dalal SS, Hanslmayr S, Staudigl T (2018). Across-subjects classification of stimulus modality from human meg high frequency activity. PLoS Comput. Biol..

[16] Ehrlich, S., Wykowska, A., Ramirez-Amaro, K. & Cheng, G. When to engage in interaction—And how? Eeg-based enhancement of robot’s ability to sense social signals in hri. In *2014 IEEE-RAS International Conference on Humanoid Robots* (2014).

[17] Monfort, M. *et al*. Moments in time dataset: One million videos for event understanding. *IEEE Transactions on Pattern Analysis and Machine Intelligence*, 1–8 (2019). 10.1109/TPAMI.2019.2901464 30802849

[18] Lee, S., Purushwalkam, S., Cogswell, M., Crandall, D. & Batra, D. Why my heads are better than one: Training a diverse ensemble of deep networks. Preprint at http://arxiv.org/abs/1511.06314 (2015).

[19] Garcia, N., Morerio, P. & Murino, V. Modality distillation with multiple stream networks for action recognition. *European Conference on Computer Vision* (2018).

[20] Garcia, N., Morerio, P. & Murino, V. Learning with privileged information via adversarial discriminative modality distillation. *IEEE Transactions on Pattern Analysis and Machine Intelligence* (2019). 10.1109/TPAMI.2019.2929038 31331879

[21] Lopez-Paz, D., Schölkopf, B., Bottou, L. & Vapnik, V. Unifying distillation and privileged information. In *International Conference on Learning Representations (ICLR)* (2016).

[22] Zhou, B., Andonian, A., Oliva, A. & Torralba, A. Temporal relational reasoning in videos. In *Proc. European Conference on Computer Vision (ECCV)*, 803–818 (2018).

[23] Lin, J., Gan, C. & Han, S. Tsm: Temporal shift module for efficient video understanding. In *Proc. IEEE International Conference on Computer Vision* (2019).

[24] Cuthbert BN, Schupp HT, Bradley MM, Birbaumer N, Lang PJ (2000). Brain potentials in affective picture processing: Covariation with autonomic arousal and affective report. Biol. Psychol..

[25] Takahashi, K. & Tsukaguchi, A. Remarks on emotion recognition from bio-potential signals. In *2nd International Conference on Autonomous Robots and Agents*, Vol. 3, 1148–1153 (2004).

[26] Chanel G, Kierkels JJM, Soleymani M, Pun T (2009). Short-term emotion assessment in a recall paradigm. Int. J. Hum Comput Stud..

[27] Koelstra S, Patras I (2013). Fusion of facial expressions and eeg for implicit affective tagging. Image Vis. Comput..

[28] Liu, S. *et al*. What makes a good movie trailer?: Interpretation from simultaneous eeg and eyetracker recording. In *Proc. 24th ACM International Conference on Multimedia*, 82–86 (ACM, 2016).

[29] Chai X, Wang Q, Zhao Y, Xin Liu O, Yongqiang L (2016). Unsupervised domain adaptation techniques based on auto-encoder for non-stationary eeg-based emotion recognition. Comput. Biol. Med..

[30] Yin Z, Wang Y, Liu L, Zhang W, Zhang J (2017). Cross-subject eeg feature selection for emotion recognition using transfer recursive feature elimination. Front. Neurorobot..

[31] Li X, Song D, Zhang P, Zhang Y, Hou Y, Bin H (2018). Exploring eeg features in cross-subject emotion recognition. Front. Neurosci..

[32] Soleymani, M., Koelstra, S., Patras, I. & Pun, T. Continuous emotion detection in response to music videos. In *Face and Gesture 2011*, 803–808 (IEEE, 2011).

[33] Zhu, Y., Wang, S. & Ji, Q. Emotion recognition from users’ eeg signals with the help of stimulus videos. In *2014 IEEE International Conference on Multimedia and Expo (ICME)*, 1–6 (IEEE, 2014).

[34] Zhu, J.-Y., Zheng, W.-L. & Lu, B.-L. Cross-subject and cross-gender emotion classification from eeg. In *World Congress on Medical Physics and Biomedical Engineering, June 7–12, 2015, Toronto, Canada*, 1188–1191 (Springer, 2015).

[35] Palazzo S, Spampinato C, Kavasidis I, Giordano D, Schmidt J, Shah M (2021). Decoding brain representations by multimodal learning of neural activity and visual features. IEEE Trans. Pattern Anal. Mach. Intell..

[36] Li R, Johansen JS, Ahmed H, Ilyevsky TV, Wilbur RB, Bharadwaj HM, Siskind JM (2021). The perils and pitfalls of block design for eeg classification experiments. IEEE Trans. Pattern Anal. Mach. Intell..

[37] Palazzo, S. *et al*. Correct block-design experiments mitigate temporal correlation bias in EEG classification. *CoRR*. http://arXiv.org/abs/2012.03849 (2020).

[38] Kavasidis, I., Palazzo, S., Spampinato, C., Giordano, D. & Shah, M. Brain2image: Converting brain signals into images. In *Proc. 25th ACM International Conference on Multimedia, MM ’17*, 1809–1817 (Association for Computing Machinery, 2017).

[39] Nishimoto S, Vu AT, Naselaris T, Benjamini Y, Yu B, Gallant JL (2011). Reconstructing visual experiences from brain activity evoked by natural movies. Curr. Biol..

[40] Barbu, A. *et al*. Seeing is worse than believing: Reading people’s minds better than computer-vision methods recognize actions. In *European Conference on Computer Vision*, 612–627 (Springer, 2014).

[41] Han J, Ji X, Xintao H, Han J, Liu T (2014). Clustering and retrieval of video shots based on natural stimulus fmri. Neurocomputing.

[42] Xintao H, Guo L, Han J, Liu T (2015). Decoding semantics categorization during natural viewing of video streams. IEEE Trans. Auton. Ment. Dev..

[43] Han J, Ji X, Xintao H, Guo L, Liu T (2015). Arousal recognition using audio-visual features and fmri-based brain response. IEEE Trans. Affect. Comput..

[44] Schalk G, McFarland DJ, Hinterberger T, Birbaumer N, Wolpaw JR (2004). Bci 2000: A general-purpose brain-computer interface (bci) system. IEEE Trans. Biomed. Eng..

[45] Blankertz B, Muller K, Curio G, Vaughan TM, Schalk G, Wolpaw JR, Schlogl A, Neuper C, Pfurtscheller G, Hinterberger T, Schroder M, Birbaumer N (2004). The bci competition 2003: Progress and perspectives in detection and discrimination of eeg single trials. IEEE Trans. Biomed. Eng..

[46] Zhao, S. & Rudzicz, F. Classifying phonological categories in imagined and articulated speech. In *2015 IEEE International Conference on Acoustics, Speech and Signal Processing (ICASSP)* (2015).

[47] Stober, S., Sternin, A., Owen, A. M. & Grahn, J. A. Towards music imagery information retrieval: Introducing the openmiir dataset of eeg recordings from music perception and imagination. In *Proc. 16th International Society for Music Information Retrieval Conference (ISMIR)* (2015).

[48] Savran, A. *et al*. Emotion detection in the loop from brain signals and facial images. In *Proceedings of the eNTERFACE 2006 Workshop* (2006).

[49] Koelstra S, Muhl C, Soleymani M, Lee J-S, Yazdani A, Ebrahimi T, Pun T, Nijholt A, Patras I (2012). Deap: A database for emotion analysis; using physiological signals. IEEE Trans. Affect. Comput..

[50] Soleymani M, Lichtenauer J, Pun T, Pantic M (2012). A multimodal database for affect recognition and implicit tagging. IEEE Trans. Affect. Comput..

[51] Zheng W-L, Bao-Liang L (2015). Investigating critical frequency bands and channels for EEG-based emotion recognition with deep neural networks. IEEE Trans. Auton. Ment. Dev..

[52] He, K., Zhang, X., Ren, S. & Sun, J. Deep residual learning for image recognition. In *2016 IEEE Conference on Computer Vision and Pattern Recognition, CVPR 2016, Las Vegas, NV, USA, June 27–30, 2016*, 770–778 (2016).

[53] Peirce JW (2007). Psychopy-psychophysics software in python. J. Neurosci. Methods.

[54] Chan A, Halgren E, Marinkovic K, Cash Sydney S (2011). Decoding word and category-specific spatiotemporal representations from meg and eeg. NeuroImage.

[55] Simanova I, van Gerven M, Oostenveld R, Hagoort P (2011). Identifying object categories from event-related eeg: Toward decoding of conceptual representations. PLoS ONE.

[56] Huettel SA, McCarthy G (2004). What is odd in the oddball task?: Prefrontal cortex is activated by dynamic changes in response strategy. Neuropsychologia.

[57] Oostenveld R, Fries P, Maris E, Schoffelen J-M (2011). Fieldtrip: Open source software for advanced analysis of meg, eeg, and invasive electrophysiological data. Comput. Intell. Neurosci..

[58] Dien J (1998). Issues in the application of the average reference: Review, critiques, and recommendations. Behav. Res. Methods Instrum. Comput..

[59] Shi, L.-C., Jiao, Y.-Y. & Lu, B.-L. Differential entropy feature for eeg-based vigilance estimation. In *2013 35th Annual International Conference of the IEEE Engineering in Medicine and Biology Society (EMBC)*, 6627–6630 (IEEE, 2013). 10.1109/EMBC.2013.6611075 24111262

[60] Hochreiter S, Schmidhuber J (1997). Long short-term memory. Neural Comput..

[61] Karim F, Majumdar S, Darabi H, Chen S (2018). Lstm fully convolutional networks for time series classification. IEEE Access.

[62] Wang, H. & Schmid, C. Action recognition with improved trajectories. In *IEEE International Conference on Computer Vision* (2013).

[63] Ioffe, S. & Szegedy, C. Batch normalization: Accelerating deep network training by reducing internal covariate shift. In *Proc. 32nd International Conference on International Conference on Machine Learning—Volume 37, ICML’15*, 448–456 (2015).

[64] Kemp B, Zwinderman AH, Tuk B, Kamphuisen HAC, Oberye JJL (2000). Analysis of a sleep-dependent neuronal feedback loop: The slow-wave microcontinuity of the eeg. IEEE Trans. Biomed. Eng..

[65] Terzano MG (2001). Atlas, rules, and recording techniques for the scoring of cyclic alternating pattern (cap) in human sleep. Sleep Med..

[66] Andrzejak RG, Schindler K, Rummel C (2012). Nonrandomness, nonlinear dependence, and nonstationarity of electroencephalographic recordings from epilepsy patients. Phys. Rev. E.

