# Supplementary material for: Understanding action concepts from videos and brain activity through subjects’ consensus
Source: Sci Rep. 2022 Nov 9;12:19073. doi: 10.1038/s41598-022-23067-2 (PMC9646846; doi:10.1038/s41598-022-23067-2)
Supplement: Supplementary file 1 — Supplementary Information. [file 41598_2022_23067_MOESM1_ESM.pdf]

Supplementary Material to Understanding Action Concepts from Videos and Brain Activity through Subjects' Consensus

October 14, 2022

A Additional details on the EEG data acquisition

Within each batch of videos showed to the participant, 3 of them were “dummy”: during dummy videos the fixation cross (which, on “regular” video is located at the center of the screen and white in color) becomes red. This should trigger the participant’s response of pressing the spacebar: we monitored, not only that the spacebar was pressed from any of the user after each dummy video, but we also monitored the reaction time for this to happen: the results, available in Table 1, show that the participants were paying a high attention to the visual stimuli so that the space bar was pressed in correspondence to a dummy video for 94.53%±8.48% of the time (on average) with an average response time of 1.20±0.89 seconds that occur after the video ends (and before the spacebar is pressed). We therefore conclude that such a sharply correct execution of this attention task is capable of guaranteeing that each participants paid a high level of attention to the visualized stimuli.

Trials were self-paced, i.e., each trial was started by the participant by pressing the spacebar key on the keyboard: between two consecutive videos, an intermediate grey screen is shown to the participant. The grey screen displays the action category name and the line “press the spacebar to start the next video”: this screen lasts until the participant pressed the spacebar. Each trial starts with 1 second inter-stimulus interval (ISI), presenting only a white fixation cross in the centre of the screen. Then, the video is presented in the centre of the screen, superimposed by the white fixation cross. All videos lasted 3 seconds. In order to maintain the focus of the participants on the videos, an oddball-like [1] task was added during the video presentation. That is, in a random order between the streaming of the videos related to one of the classes of interest, 3 dummy videos where displayed. During the presentation of the dummy videos (3 for each category) the white cross turned red for 250 ms. This colour change was happening at a random moment between 1500 and 3000 ms after the beginning of the video. Participants were asked to press the spacebar as fast as possible when they noticed the colour change. These dummy trials were subsequently removed during the EEG analysis phase.

In addition to a careful acquisition stage, we also took advantage of an established pre-processing technique to make sure that the EEG data convey data regarding the visual stimuli: baseline removal. In order to explain why baseline removal can actually help in this respect, let us point out that one second of time passes after the class name is disclosed to the participant and before the video start. Given our efforts in preserving the participant’s attention towards the video screen, we conjecture that, during this second the participant is abstractly thinking about the action’s category that will be soon displayed on the video. In other words, in this first second, we are capturing the pure conceptual mental activity of each of the participant when he/she abstractly imagine the class whose semantic label has been disclosed. We use the average EEG activity related to this 1 second segment as the baseline that we adopt

afterwards for the pre-processing. Mathematically, we subtract each element of the time series corresponding to the EEG data concurrent to the video by this baseline: this means that we remove the average neural activity related to an exclusive conceptual mentalization of a given action class, so that, the residual EEG activity that results from this operation encapsulates video-related visual activity, cleaned of category-related abstract thinking.

	S1	S2	S3	S4	S5	S6	S7	S8	S9	S10	S11
acc [%]	100	100	100	100	80.00	100	100	86.67	100	86.67	93.33
$\max_t$ [s]	1.13	0.63	1.11	1.90	1.40	0.68	0.63	1.42	0.75	1.25	6.11

	S12	S13	S14	S15	S16	S17	S18	S19	S20	S21	S22
acc [%]	93.33	86.67	100	100	73.33	100	66.67	86.67	100	100	100
$\max_t$ [s]	0.85	0.75	1.13	0.98	1.28	0.78	1.45	1.3	0.99	1.22	1.10

	S23	S24	S25	S26	S27	S28	S29	S30	S31	S32	S33
acc [%]	100	80.00	100	80.00	73.33	100	100	93.33	100	100	100
$\max_t$ [s]	0.63	0.67	3.42	0.80	1.08	0.67	0.68	1.40	0.95	0.65	1.05

	S34	S35	S36	S37	S38	S39	S40	S41	S42	S43	S44
acc [%]	100	93.33	93.33	100	100	93.33	86.67	100	100	93.33	100
$\max_t$ [s]	0.65	2.25	0.65	1.38	0.85	0.77	0.75	0.67	0.77	1.30	0.72

	S45	S46	S47	S48	S49	S50	**AVG**	**std**
acc [%]	100	93.33	93.33	100	100	100	**94.53**	**±8.48**
$\max_t$ [s]	1.00	2.18	1.25	1.57	0.88	1.29	**1.20**	**±0.89**

Table 1: For each of the 50 participants (referred as S followed by a progressive number in the range {1,..,50}), we report two quantitative indicators to monitor their correct accomplishment of our adopted oddball-like task (fixation cross changing color). We report the accuracy with which the space bar is pressed each time a dummy video is shown ("acc"), expressing such value as a percentage. We also provide the maximal response time that was taken by each single subject to press the space bar, while considering all dummy videos he was shown (this value is referred as "$\max_t$" and it is espressed in seconds. For a comprehensive evaluation, we provide also the average and the standard deviation for the acc and $\max_t$ values (in bold).

A.1 Instructions for the participants

Here we list the exact instructions communicated to the participants before the acquisition and before each block.

```
START INSTRUCTIONS:
You will be asked to perform two tasks during this experiment:
You will see a series of videos. For each video,
please rate how much it represents a certain action by using
the slider after the video.
During the video, you also have to keep your hand on the keyboard,
```

Vanilla CNN Vanilla LSTM Two-branch LSTM (with attention)

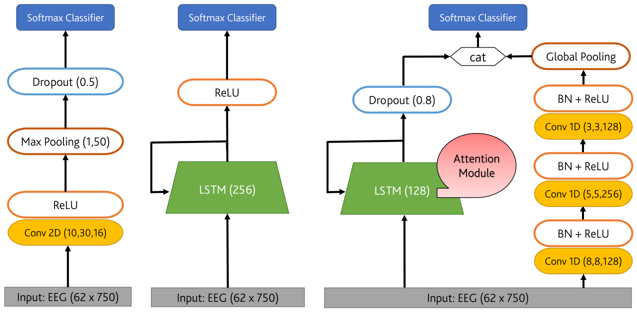

Figure 1: Deep learning architectures used to learn features from EEG data.

```
as the fixation cross will become red in some videos.
Please press the spacebar as soon as you notice this change.
At the end of each block, you will be given feedback
about how many changes you noticed.

BLOCK INSTRUCTIONS: (before each block)
Now you will see some examples of the action XXX.
Please rate how much each video represents this action
by using the slider after the video.
```

B Visualization of the architectures used for EEG data

In Figure 1, we show the architectures processing EEG data, described in the Methods section.

C An analysis of the performance of EEG images.

As proposed in [2], EEG images are an effective strategy to cast EEG input data into color images (by converting theta, alpha and beta frequency band into the R, G and B color channels of an image – see Methods. Once EEG data is casted into image-like input stream, convolutional neural networks can be adopted, such as ResNet-50. In this paragraph, we are interested in analyzing the performance of this model in terms of the ability of confusing similar action classes.

We provide the Receiving Operator Characteristic (ROC) curves by computing the related area under it (AUC). To do so, we extract the softmax scores

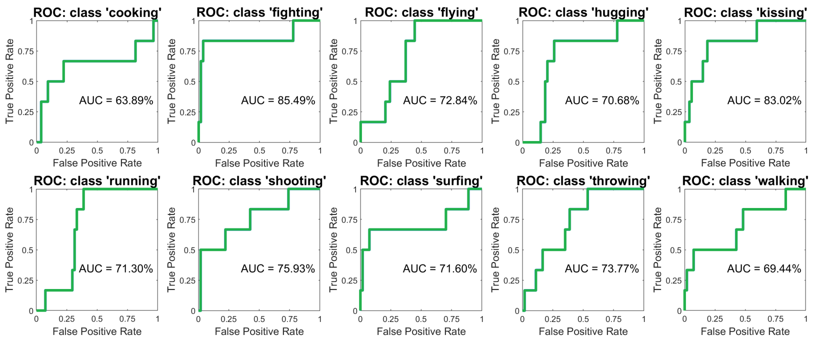

Figure 2: Receiving Operator Characteristic (ROC) curve and the relative area under it (AUC), relative to the ResNet-50 model fed with EEG images.

from our model trained on EEG images, we compare the scores with which any of the test videos from our dataset is associated by the model to each of the categories, while also having access to the ground truth label. The ROC curves, for each of the 10 classes of our dataset, and the relative AUC values are reported in Figure 2. These indicators are useful in spotting which actions are easier/harder to recognize in absolute terms: *fighting* seems the easier one (AUC = 85.49%), together with *kissing* (83.02%). Actions such as *flying, hugging, running, shooting, surfing* or *throwing* are "intermediate" since their respective AUC is above 70%. Even, for the most difficult actions (walking - AUC = 69.44% and cooking - AUC = 63.89%), the classification scores are still reliable enough to certify that the task of recognizing implicit actions from video can be tackled and solved with a sufficient degree of success.

Global statistics of the classification performance can be found in Figure 4 to compare the predictions made by the EEG images + ResNet50 models with the ground truth. As expected, actions such as *kissing* and *hugging* have a high chance to be confused since both of them imply close physical interaction between two human agents, and therefore the visual cues which help in implicitly referring to these two actions are highly overlapping Similarly, *walking* and *running* are confused for the very same reason: the most likely visual cue that helps in disambiguating these two actions is the execution speed, and it may be actually subjective in the way a running/walking action is implicitly referred in a video. *Flying* and *throwing* are sometimes confused between each others, and this is understandable from the fact that they both refer to the case in which something is displacing "in the air". In all other cases, the remaining actions are quite well classified, as another evidence for the fact that EEG is a reliable data modality for the sake of recognizing actions, even when they are only implicitly referred in a video and not explicitly visualized.

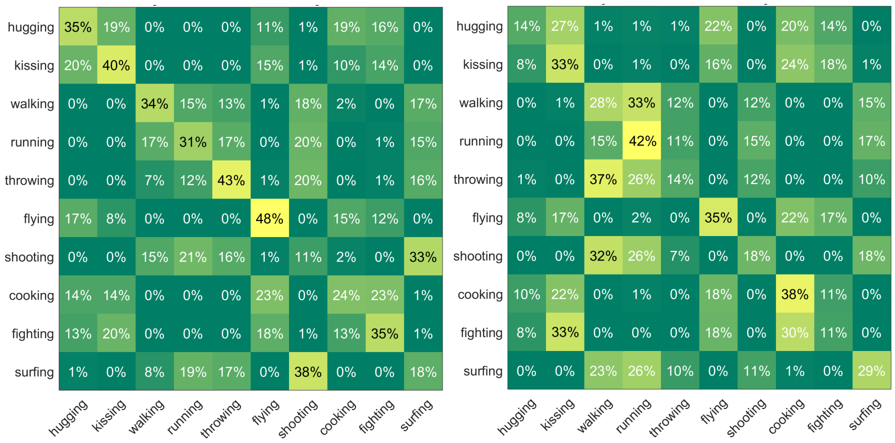

Figure 3: Comparison of confusion matrices obtained using two different computational approaches. *Left*: DE+MLP, a multi-layer perceptron (MLP) fed with differential entropy (DE) features (*left*). *Right:* the attention-based LSTM neural network proposed by [Karim et al. 2018]. In both cases (*left* and *right*), we list ground truth (actual) classes by row and predictions by columns.

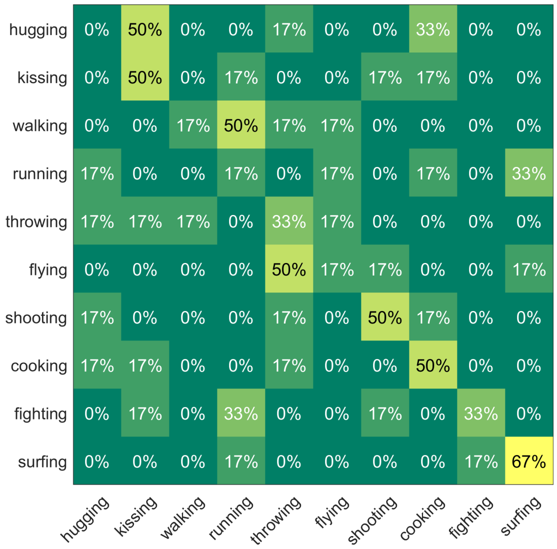

Figure 4: Confusion matrix related to the ResNet-50 model trained on EEG images. Actual classes are listed by rows, while predictions are displayed by columns.

Bibliography

[1] Scott A Huettel and Gregory McCarthy. What is odd in the oddball task?: Prefrontal cortex is activated by dynamic changes in response strategy. *Neuropsychologia*, 42(3):379–386, 2004.

[2] Pouya Bashivan, Irina Rish, Mohammed Yeasin, and Noel Codella. Learning representations from eeg with deep recurrent-convolutional neural networks. *Internetional Confernece on Learning Representations (ICLR)*, 2016.
